# Predictors of physicians’ intentions to use clinical practice guidelines on antimicrobial in tertiary general hospitals of China: a structural equation modeling approach

**DOI:** 10.1186/s13756-021-00966-z

**Published:** 2021-06-30

**Authors:** Qingwen Deng, Zhichao Zeng, Yuhang Zheng, Junhong Lu, Wenbin Liu

**Affiliations:** School of Public Health, Fujian Medical University, Room 108 in the Building for School of Public Health, No.1 Xuefubei Road, Minhou District, Fuzhou, 350122 China

**Keywords:** Antimicrobials, Clinical practice guidelines, Structural equation modeling, Utilization, China

## Abstract

**Background:**

With inappropriate use of antimicrobials becoming a great public health concern globally, the issue of applying clinical practice guidelines (CPGs) to regulate the rational use of antimicrobials has attracted increasing attention. Taking tertiary general hospitals in China for example, this study aimed to identify factors to investigate the comprehensive influencing mechanism for physicians’ intention to use CPGs on antimicrobials.

**Methods:**

Based on the integration of Theory of Planned Behavior (TPB), Technology Acceptance Model (TAM), and Technology-Organization-Environment framework (TOE), a questionnaire survey was conducted covering potential determinants of affecting physicians’ intentions to use CPGs on antimicrobials at the individual level (attitude, subjective norms and perceived risk), technical level (relative advantage and ease of use), and organizational level (top management support and organizational implementation). Data were collected from 644 physicians in tertiary general hospitals in eastern, central and western China, which were obtained by multi-stage random sampling. The structural equation modeling (SEM) was used to link three-level factors with physicians’ behavioral intentions.

**Results:**

The majority of the participants (94.57%) showed a positive tendency toward intention to use CPGs on antimicrobials. The reliability and validity analysis showed the questionnaire developed from the theoretical model was acceptable. SEM results revealed physicians’ intentions to use CPGs on antimicrobials was associated with attitude (*β* = 0.166, *p* < 0.05), subjective norms (*β* = 0.244, *p* < 0.05), perceived risk (*β* = − 0.113, *p* < 0.05), relative advantage (*β* = 0.307, *p* < 0.01), top management support (*β* = 0.200, *p* < 0.05) and organizational implementation (*β* = 0.176, *p* < 0.05). Besides, subjective norms, perceived risk, relative advantage, ease of use, and top management support showed their mediating effects from large to small on the intentions, which were 0.215, 0.140, 0.103, 0.088, − 0.020, respectively.

**Conclusions:**

This study revealed the significance of multifaceted factors to enhance the intention to use CPGs on antimicrobials. These findings will not only contribute to the development of targeted intervention strategies on promoting the use of CPGs on antimicrobials, but also provide insights for future studies about physicians’ adoption behaviors on certain health services or products.

**Supplementary information:**

The online version contains supplementary material available at 10.1186/s13756-021-00966-z.

## Background

Antimicrobials, which have been critically important in the evolution of medical treatment, effectively reduces the morbidity and mortality from infections [1]. However, the spread of inappropriate use of antimicrobials has driven the emergence of antimicrobial resistance (AMR), which is a widely acknowledged threat to global health and sustainable development [2]. It leads to weakened effectiveness and persistent infections [3] that will greatly undermine our ability to fight infectious disease, resulting in negative consequences at the individual and societal levels [4], such as longer hospital stays, higher medical costs, poor patient outcomes and waste of health resources. More than 20 billion dollars in the American health system and over 700,000 deaths worldwide were linked to AMR in 2016 [5]. The World Bank estimates that AMR might put 28 million people in an extremely poor situation by 2050, and loss of the global gross domestic product caused by AMR would be close to the 2008 global financial crisis [6].

To avoid further deteriorations caused by AMR and improve outcomes of antimicrobials, many countries have launched clinical practice guidelines (CPGs) on antimicrobials, which provide recommendations for physicians based on the current best evidence. As reported by the American Institute of Medicine [7], CPGs have played an important role in standardizing clinical treatment behaviors, improving the quality of medical services, and promoting patients’ health. Although clear principles had been established with significant effectiveness of CPGs well proved, the expansion of regulation implementation was still halted with poor adherence to regarding guidelines. This situation was outstanding especially in many developing countries, where antimicrobials consumption doubled between 2000 and 2015 [8]. For instance, in China, one of the world’s largest consumers of antimicrobials for human health [9], the inappropriate use of antimicrobials is still striking even after the launch of Guiding Principles for Clinical Application of Antimicrobials in 2015 [10].

Given the severe situation of AMR worldwide and guidelines’ contributing role of improving service quality and patients’ health, the key point of improving the AMR issue is that the appropriate use of antimicrobials could be regularized by the use of CPGs on antimicrobials [11]. Efforts have been made to understand which factors could predict physicians’ antimicrobials prescribing using various behavioral theories, such as the Theory of Planned Behavior (TPB), the knowledge-attitude-practice (KAP) model, the Technology Acceptance Model (TAM), and the Technology-Organization-Environment framework (TOE). The focus of these theories varies from the individual to the technical to the organizational environment. However, despite a few studies have considered the influencing factors from different levels of prescribing behavior [2], most of the current studies were fragmented and focused on only one aspect of the determinants. For example, many studies have investigated health professionals’ beliefs and practices from the individual level [12–16]. And more importantly, our understanding on physicians’ actual intentions of using CPGs on antimicrobials and its influencing factors is still limited. As key stakeholders in clinical practice, it’s necessary to understand physicians’ beliefs and uses of CPGs on antimicrobials and reasons for using or not using it if we did attempt to promote the appropriate use of antimicrobials [1, 17]. While to some extent, behavioral intention figures a proxy role on actual behaviors [18]. If individuals show a positive or negative intention, we presume that they would tend to use or not use a specified technology or other products. Therefore, we targeted physicians in tertiary general hospitals, aimed to establish a model that integrated from TPB, TAM and TOE for determining physicians’ intentions to use CPGs on antimicrobials and its influencing factors. The findings can not only serve as evidence to better AMR control via the promotion of the use of CPGs on antimicrobials, but also provide a feasible reference for future research on the influencing factors of physicians’ intention or behaviors on utilizing certain health services or products.

## Methods

### Study setting

This study was conducted in tertiary general hospitals in China. Although the regulation policy for the clinical application of antimicrobials covers all levels of medical institutions, for the weakness of the primary medical services system in China, the provision of vast medical services is heavily dependent on hospitals, especially tertiary hospitals. In 2019, tertiary hospitals received 1.77 billion medical visits [19]. As one of the major consumers of antimicrobials, the irrational use of antimicrobials in tertiary hospitals is quite prominent. Thus, it’s necessary to regulate the use of antimicrobials of physicians in tertiary hospitals for reducing AMR.

### Theoretical framework

**Fig. 1:**
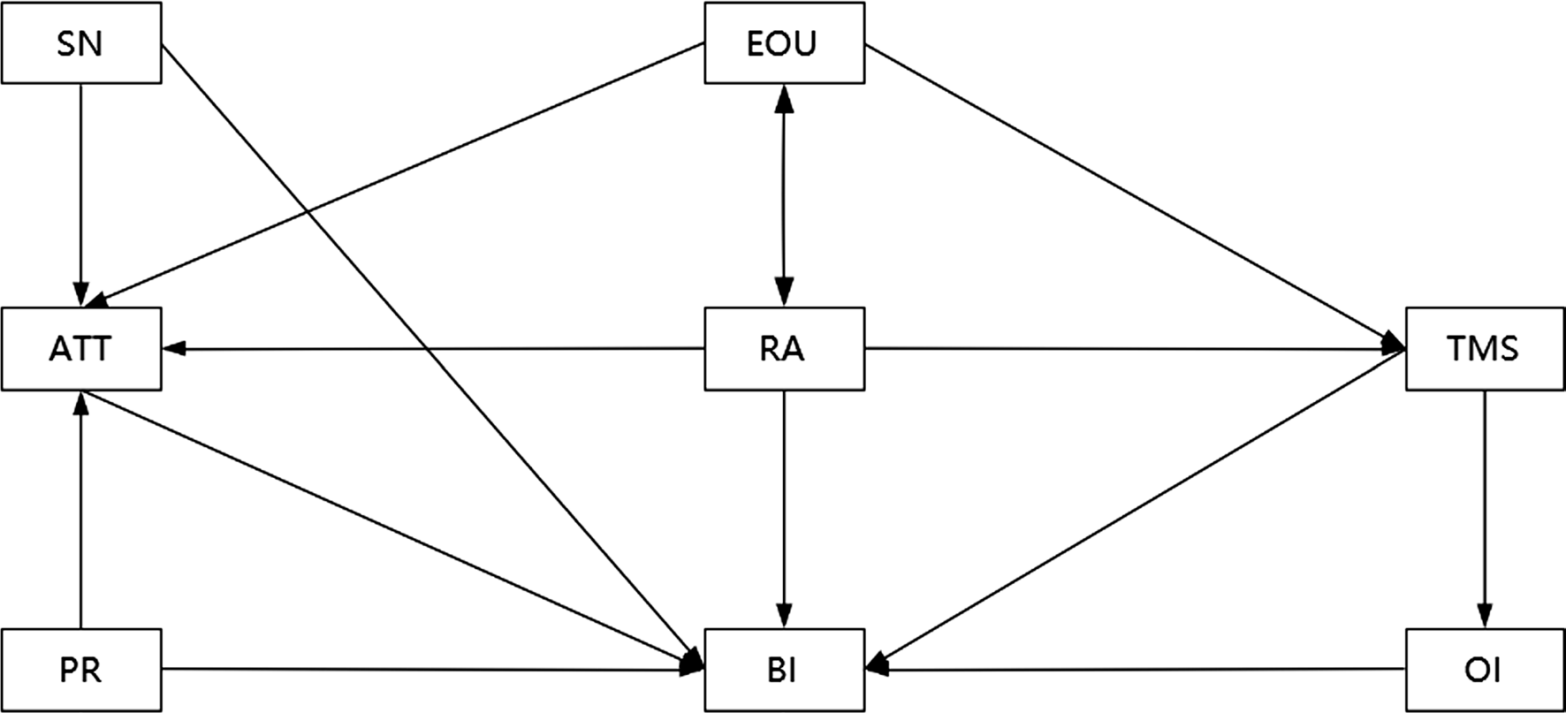
The theoretical framework

The theoretical framework (Fig. 1) was adapted from the integration of TPB, TAM and TOE to illuminate the determinants of physicians’ intentions to use CPGs on antimicrobial from three levels, namely individual level (physicians), technical level (CPGs on antimicrobial), and organizational level (hospitals).

### Individual-level factors

Proposed by TPB, behavioral intention is a function of three factors, including attitude, subjective norms and perceived behavioral control [20]. Attitude is defined as a positive or negative evaluation of a particular behavior [21], many studies showed a strong correlation between attitude and intention [22, 23]. Subjective norms are kinds of perceived criteria and social pressure to engage or not to engage in behavior [24], which may also significantly affect physicians’ intentions. Also, perceived behavioral control reflects the person’s belief that an action is under his or her control, such as perceived risk. Risk perception is associated with low intentions.

### Technical-level factors

Proposed by TAM, the relative advantage is a degree to which new technology or product is more advantageous than the original or competing ones [25], while ease of use is a degree to which the potential user expects the product can perform with ease [26, 27]. Regarding the intentions to use CPGs, the physicians and managers are more inclined to adopt the guidelines having better outcomes and efficiency with no additional effort and time to learn how to implement.

### Organizational-level factors

TOE suggests top management support “can foster innovation by creating an organizational context that welcomes change and is supportive of innovations” [28]. In hospitals, top management’s involvement in the use of CPGs on antimicrobials through formal measures (e.g. funding, training, and system building), can ensure the accomplishment of intended outcomes to a great extent [29]. Organizational implementation refers to the whole implementation process of CPGs on antimicrobials, including providing relevant information, supervision, and inspection, corrective feedback, respectively [30, 31].

### Measurements

Based on the theoretical model, as well as the literature review of previous studies, a questionnaire with 30 items was developed for this study (Additional file 1). Three items in Part 1 were used to measure the intentions to use CPGs on antimicrobials of physicians. There were 21 items in Part 2, covering seven potential factors: attitude, subjective norms, perceived risk, relative advantage, ease of use, top management support and organizational implementation. Each item in Part 1&2 corresponding to the constructs was measured using a five-point Likert scale, where 1 = Strongly disagree, 2 = Disagree, 3 = Neutral, 4 = Agree, and 5 = Strongly agree. And Part 3 was a personal information card consisted of 6 items, including several basic characteristics of participants like gender, age, education, professional degree, department, and years in practice.

### Sampling

Considering the diverse level of socio-economic development in different regions of China, a cross-sectional survey was conducted using a multistage sampling strategy. Firstly, Fujian, Hubei, Yunnan & Sichuan provinces were randomly selected respectively on behalf of eastern, central and western regions of China. Secondly, 4 tertiary general hospitals were selected from each of the selected regions. Lastly, in each selected hospital, 16–20 physicians were randomly sampled from major departments of internal medicine and surgery, respectively. And 3–5 physicians were randomly sampled from departments of gynecology and obstetrics, ophthalmology and otorhinolaryngology, orthopedics, and others, respectively. Thus, 50–60 physicians from each hospital were invited to participate in the survey.

### Data collection

A cross-sectional questionnaire survey was performed to investigate the determinants of physicians’ intentions to use CPGs on antimicrobials in China. With the support of sampled hospitals, each round for filling out the questionnaire was accompanied by trained facilitators. The purpose of the study and the use of data will be explained in detail to ensure the participants understand what they needed to do and how to do it. All responses were anonymous, filled out by the participants at their convenience and returned directly to the researchers.

Data collection started from April 2018 and lasted for nearly one year. Overall, a total of 676 questionnaires were returned. After excluding responses that (1) provided the same response for all items, (2) incomplete questionnaires, we obtained 644 valid questionnaires with a valid response rate of 95.27%.

### Data analysis

This study used SPSS 21.0 and AMOS 17.1 software programs as the two main statistical tools to analyze the data. To analyze the descriptive data and investigated variables clearly, several steps were followed. Firstly, descriptive statistics were performed for the analysis of participants’ distribution characteristics. Secondly, we conducted the assessment of reliability and validity via Cronbach’s *α* Coefficient and factor analysis to tell whether the questionnaire was acceptable. Finally, structural equation modeling (SEM) was used to analyze the mechanism and the relationship between the factors via path analysis and mediating effect test. The path coefficients calculated by path analysis are equivalent to the standardized regression coefficients and direct effects. The mediating effects (indirect effects) and total effects were obtained by mediating effect test. The indirect effects refer to the influence of one variable on another through a third variable, and its value was calculated through the Bootstrap method. If the value does not contain zero in its 95% confidence interval, the mediating effect is considered significant [32].

## Results

### Descriptive characteristics

A total of 644 physicians were included in this study. Among the participants, 54.50% (n = 351) were males and 45.50% (n = 293) were females. Most participants were in the age group of under 35 years old (55.75%, n = 359), followed by 35–44 years old (34.63%, n = 223). In terms of educational level, 98.91% (n = 637) reported having a bachelor’s degree or above. The proportion of the participants with the professional titles of junior, intermediate, senior was 38.82%, 38.35%, 22.83%, respectively. Nearly 90% of the participants had less than 15 years of practice experience. And about a third of participants were from the region of the east, central and west, respectively (Table 1).

**Table 1:** Demographic characteristics of participants

Variable	Category	Frequency	Percentage (%)
Gender	Male	351	54.50
	Female	293	45.50
Age	< 35 years old	359	55.75
	35–44 years old	223	34.63
	≥ 45 years old	62	9.63
Education	Junior college or below	7	1.09
	Bachelor	218	33.85
	Master	342	53.11
	Doctor	77	11.96
Professional title	Junior	250	38.82
	Intermediate	247	38.35
	Senior	147	22.83
Department	Internal medicine	233	36.18
	Surgery	188	29.19
	Gynecology and obstetrics	57	8.85
	Ophthalmology and otorhinolaryngology	65	10.09
	Orthopedics	44	6.83
	Other	57	8.85
Years in practice	< 5 years	225	34.94
	5–10 years	192	29.81
	11–15 years	162	25.16
	16–20 years	59	9.16
	> 20 years	6	0.93
Region	East	217	33.70
	Central	210	32.61
	West	217	33.70

### Reliability and validity

Table 2 reports Cronbach’s alpha, composite reliability (CR), and the average variance extracted (AVE) of all constructs. The Cronbach’s alpha of 8 constructs and the whole questionnaire were all greater than the recommended threshold of 0.7 [33], ranging from 0.810 to 0.885, suggesting internal consistency can be considered adequate. Besides, all factor loading values of items were above the acceptability value of 0.5 [34]. Moreover, the CR scores and AVE values of all constructs were above the recommended value of 0.7 [35] and 0.5 [36], respectively, which indicated a good convergent validity.

**Table 2:** Results of reliability and convergent validity analyses

Construct	Item	Factor loading	Cronbach’s α	AVE	CR
Attitude	ATT1	0.712	0.862	0.632	0.837
	ATT2	0.808			
	ATT3	0.858			
Subjective norms	SN1	0.776	0.876	0.707	0.879
	SN2	0.864			
	SN3	0.879			
Perceived risk	PR1	0.699	0.810	0.593	0.813
	PR2	0.808			
	PR3	0.799			
Behavioral intention	BI1	0.766	0.859	0.610	0.824
	BI2	0.810			
	BI3	0.766			
Relative advantage	RA1	0.779	0.854	0.666	0.857
	RA2	0.835			
	RA3	0.833			
Ease of use	EOU1	0.806	0.869	0.692	0.870
	EOU2	0.865			
	EOU3	0.823			
Top management support	TMS1	0.785	0.837	0.627	0.834
	TMS2	0.745			
	TMS3	0.842			
Organizational implementation	OI1	0.839	0.885	0.728	0.889
	OI2	0.898			
	OI3	0.821			
The whole questionnaire			0.885		

Then, we followed Fornel and Larcker’s (1981) suggestion [34] to calculate the square root of AVE. As shown in Table 3, the square root of AVE (reported in the diagonal of correlation matrix) of each construct is higher than the correlation coefficients of any construct with other constructs, which means the discriminant validity is acceptable.

**Table 3:** Results of discriminant validity analysis

Construct	PR	SN	EOU	RA	TMS	ATT	OI	BI
PR	0.770							
SN	0.000	0.841						
EOU	0.000	0.000	0.832					
RA	0.000	0.000	0.671	0.816				
TMS	0.000	0.000	0.588	0.644	0.792			
ATT	− 0.119	0.619	0.198	0.335	0.208	0.795		
OI	0.000	0.000	0.468	0.513	0.597	0.166	0.853	
BI	− 0.133	0.347	0.438	0.581	0.571	0.504	0.519	0.781

### Intentions to use CPGs on antimicrobials and measurement scores of participants

A high intention to use CPGs on antimicrobial in clinical practice (Mean = 4.12, SD = 0.58) were evident (Table 4). The overwhelming majority (94.57%) scored above neutral, and 33.39% of the intention scores were greater than average. The attitudes of the participants showed a strong tendency in favor of the use of CPGs on antimicrobials (M = 4.29, SD = 0.56). Their perceived pressure (subjective norms) from influential people was relatively high (Mean = 4.16, SD = 0.59), while their perceived risk of using CPGs on antimicrobials was found to be low (Mean = 2.23, SD = 0.85). Most participants, specifically, 87.42% and 84.16% felt positive in relative advantage (Mean = 3.96, SD = 0.67) and ease of use (Mean = 3.84, SD = 0.66), respectively. The scores of top management support (Mean = 4.01, SD = 0.61) and organizational implementation (Mean = 4.05, SD = 0.60) portrayed the participants’ appreciation of the organizations’ readiness to use CPGs on antimicrobials.

**Table 4:** Measurement scores of the participants

Measurements	Mean	SD	Skewness	Median	N (%) of scores > 3
Intention	4.12	0.58	− 0.489	4	609 (94.57)
Attitude	4.29	0.56	− 0.268	4	625 (97.05)
Subjective norms	4.16	0.59	− 0.250	4	601 (93.32)
Perceived risk	2.23	0.85	0.700	2	89 (13.82)
Relative advantage	3.96	0.67	− 0.107	4	563 (87.42)
Ease of use	3.84	0.66	− 0.229	4	542 (84.16)
top management support	4.01	0.61	− 0.312	4	587 (91.15)
organizational implementation	4.05	0.60	− 0.275	4	591 (91.77)

*SD* Standard deviation

### Structural equation modeling

A favorable fitness of data into the theoretical framework was found: *χ*^2^/df = 3.736 (< 5), GFI = 0.900 (> 0.9), AGFI = 0.873 (> 0.85), CFI = 0.933 (> 0.9), NFI = 0.911 (> 0.9), IFI = 0.933 (> 0.9), RMSEA = 0.065 (< 0.08), which demonstrated the research model has fit the data well.

**Fig. 2:**
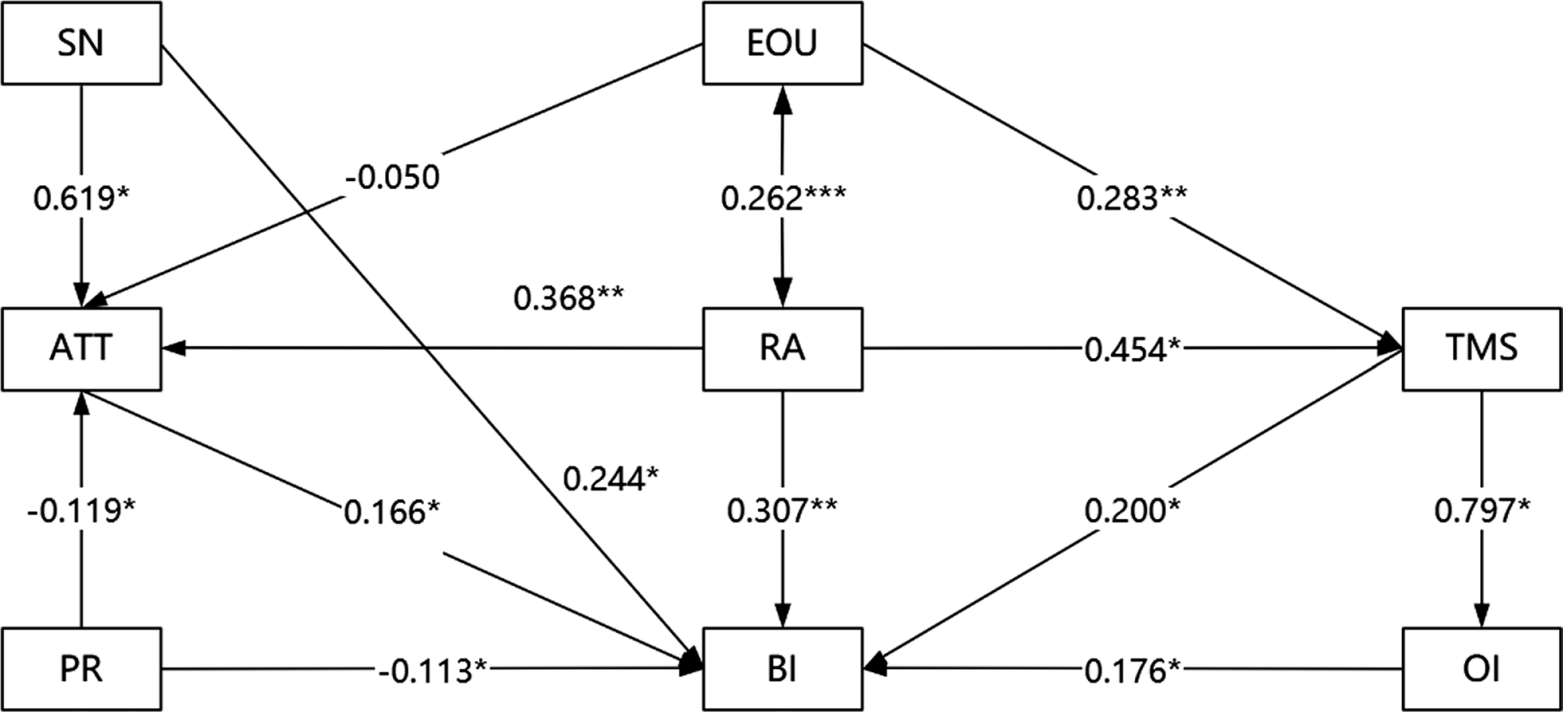
Determinants of physicians’ intentions to use CPGs on antimicrobials. *p < 0.05; **p < 0.01; ***p < 0.001

The final structural model with the standardized estimates among the constructs is presented in Fig. 2 and Table 5. Totally 78.89% of the variance was explained by the model. Regarding the determinants of physicians’ intentions to use CPGs on antimicrobials, the model indicated that, at the individual level, an attitude in favor of CPGs on antimicrobials was associated with higher intentions to use CPGs on antimicrobials (β = 0.166, *p* < 0.05). Subjective norms predicted physicians’ intentions to use CPGs on antimicrobials (β = 0.244, *p* < 0.05). Greater perceived obstacles and risks were linked to lower intentions to use CPGs on antimicrobials (β =—0.113, *p* < 0.05). At the technical level, better performance in relative advantage was associated with higher intentions to use CPGs on antimicrobials (β = 0.307, *p* < 0.01), while the impact of ease of use on attitude toward CPGs on antimicrobials was not significant (β =—0.050, *p* > 0.05), and the direct influence of ease of use on the intentions to use CPGs on antimicrobials was not found. At organizational-level, top management support (β = 0.200, *p* < 0.05) and organizational implementation (β = 0.176, *p* < 0.05) were linked to stronger intentions to use CPGs on antimicrobials.

**Table 5:** Results of standardized direct, indirect, and total effects

Paths	Direct effects (path coefficients)	Indirect effects	Total effects
Attitude → Behavioral intention	0.166*	0	0.166*
Subjective norms → Attitude	0.619*	0	0.619*
Subjective norms → Behavioral intention	0.244*	0.103**	0.347**
Perceived risk → Attitude	− 0.119*	0	− 0.119*
Perceived risk → Behavioral intention	− 0.113*	− 0.020*	− 0.133*
Relative advantage → Attitude	0.368**	0	0.368*
Relative advantage → Top management support	0.454*	0	0.454*
Relative advantage → Behavioral intention	0.307**	0.215*	0.522**
Ease of use → Attitude	− 0.050	0	− 0.050
Ease of use → Top management support	0.283**	0	0.283**
Ease of use → Behavioral intention	–	0.088**	0.088**
Top management support → Organizational implementation	0.797*	0	0.797*
Top management support → Behavioral intention	0.200*	0.140*	0.340*
Organizational implementation → Behavioral intention	0.176*	0	0.176*

*p < 0.05; **p < 0.01

On the whole, relative advantage figured the strongest direct and total effects on intention (0.307/0.522), followed by subjective norms (0.244/0.347) and top management support (0.200/0.340). Additionally, significant mediating effects were also found in the model. Except that attitude and organizational implementation have no mediating effect on behavioral intention, other constructs mediated the significant effects on the relationship between them. Among them, relative advantage exerted the strongest indirect effects on intention (0.215), followed by top management support (0.140), subjective norms (0.103), ease of use (0.088) and perceived risk (− 0.020).

## Discussion

### Main findings

This study revealed that physicians in tertiary general hospitals of China have high intentions to use CPGs on antimicrobials, with relatively favorable evaluations and perceptions on CPGs on antimicrobials. The integrated model of TPB, TAM and TOE fits well with the data: intentions to use CPGs on antimicrobials are directly or indirectly predicted by the attitudes, subjective norms, perceived risk, relative advantage, ease of use, top management support and organizational implementation.

### Comparison with other studies

#### Effects of individual-level factors

Consistent with previous studies [37–41], attitude and subjective norms are important factors that have a direct positive influence on physicians’ intentions to use CPGs on antimicrobials. In the field of health care, the attitude and subjective norms of health professionals are often highlighted as they related to a sense of security in specific behaviors [42], and are shaped in part by perceived external pressures on them. Perceived risk has a negative impact on physicians’ intentions to use CPGs on antimicrobials. The assumption and perception of various potential risks (such as income reduction, failure in disease control, and patient dissatisfaction) in the implementation process may hinder the occurrence of behaviors. In addition to the direct impacts, both subjective norms and perceived risk have indirect effects on intentions through attitude. Thus, we need to pay attention to the various linking effects of other associated factors if we want to improve the effect of attitude on intentions.

#### Effects of technical-level factors

Relative advantage is directly associated with attitude and intention, which is also in accordance with previous research [43, 44]. Meanwhile, relative advantage figured the strongest total, direct and indirect effects on intention, which revealed that physicians’ intentions to use CPGs on antimicrobials can be greatly strengthened if the CPGs on antimicrobials have advantages in improving clinical efficacy, promoting practice efficiency and ensuring patients’ safety.

In contrast to expectations, ease of use was not shown to have a significant influence on attitude, which is different from the findings of Davis and Venkatesh [45, 46]. The plausible reason may be that the role of ease of use often has no significant effect at the beginning of implementation, which is similar to the statement proposed in a previous study [47] that the impact of ease of use is limit at an early stage of technology uptake. Another possibility we also can’t rule out is that the impact of ease of use on attitude was reduced by relative advantage, which is also included in the final model demonstrating a great association with ease of use and significant impacts on attitude.

#### Effects of organizational-level factors

In terms of hospital level, it is reported that physicians’ intentions and utilization of using CPGs on antimicrobials were significantly influenced by top management support [48]. As influential people in the hospitals, top managers play important roles in developing the organizational vision and culture, as well as shaping the expected behavior and norms of physicians [49]. The support of hospital top managers for the use of CPGs on antimicrobials is often reflected in the establishment of a series of systems and mechanisms to assess, motivate and supervise the realization of corresponding goals, which can not only directly stimulate the use intentions of physicians, but also have an indirect effect on the intentions through organizational implementation [50]. In addition to the institutional design, the overall application of CPGs on antimicrobials in hospitals also change intentions and further affect the practical use of physicians. To some extent, top management support and organizational implementation act as external social norms, exerting direct or indirect influences on physicians’ behaviors from the outside to the inside, until they gradually adjust their behaviors to be consistent with the organization [51].

### Policy implications

Based on the understandings of the influencing mechanism of physicians’ intentions to use CPGs on antimicrobials, several intervention strategies can be highlighted for further improving physicians’ intentions and practical use of CPGs on antimicrobials.

For hospital managers, the use of CPGs on antimicrobials by physicians is promoted more through explicit means such as regulations. Firstly, providing specific support (e.g., funding, personnel, information, system) is one of the effective measures to promote physicians’ intentions to use CPGs on antimicrobials. Secondly, the establishment of feedback and expert panel is indispensable to timely solve various issues of the implementation of CPGs on antimicrobials. Thirdly, education and training are essential for increasing the knowledge of CPGs on antimicrobials (including the perceptions of usefulness, ease of use, and so on), as well as to help physicians foster positive attitudes toward CPGs on antimicrobials and its use, which can be adopted as long-term strategies [2].

Hospital managers can also advance the use of CPGs on antimicrobials by physicians in an implicit approach, that is, by leveraging subjective norms to increase physicians’ intentions to use CPGs on antimicrobials. Specifically, this means mobilizing influential people for physicians (generally referred to department directors, authoritative experts, etc.) to adapt physicians’ compliance with CPGs on antimicrobials, which is an internalized process.

### Strengths and limitations

To the best of our knowledge, there were very few national surveys to investigate the related behaviors of CPGs on antimicrobials among physicians, particularly in developing countries. This study examined the mechanism of physicians’ intentions to use CPGs on antimicrobials based on the integration of TPB, TAM and TOE, which allowed us to systematically consider the factors associated with the intentions to use CPGs on antimicrobials from three levels, namely individual, technical and organizational determinants.

There are also some limitations to this study. Firstly, all the data were obtained by self-reported, we cannot rule out the social desirability bias [52] that some physicians may be unwilling to voice negative assessments about themselves and the hospitals. Secondly, limited to time and fund, we focus the factors at the individual, technical, and organizational-level in this study, and the influence of external environmental factors will be explored in future research. Thirdly, considering the limitation of a cross-sectional study in causality interpretations, future research may involve sample at different point of time to form a panel data, which will be more robust in capturing the influencing factors.

## Conclusions

The present study investigated the determinants of the physicians’ intentions to the utilization of CPGs on antimicrobials. SEM approach was used to verify the proposed conceptual research framework. The findings of this study revealed the significance of multifaceted factors to enhance the intention to use CPGs on antimicrobials, including attitude, subjective norms, perceived risk, relative advantage, ease of use, top management support, and organizational implementation. These findings will not only benefit tailoring future interventions for expanding the utilization of CPGs on antimicrobials, but also provide clues for future research about physicians’ adoption behaviors on certain health services or products.

## Supplementary information

**Additional file 1.** Questionnaire. The questionnaire represents the data collection instrument that was developed for this study, hasn’t previously been published elsewhere.

**Additional file 2.** STROBE_checklist_cross-sectional. The checklist represents the details of the manuscript, which reports the information that meets the criteria of STROBE guidelines.

## Data Availability

The datasets generated during and/or analyzed during the current study are available from the corresponding author on reasonable request.

## Abbreviations

AMR: Antimicrobial resistance
CPGs: Clinical practice guidelines
SEM: Structural equation modeling
