# Supplementary material for: Predictors of physicians’ intentions to use clinical practice guidelines on antimicrobial in tertiary general hospitals of China: a structural equation modeling approach
Source: Antimicrob Resist Infect Control. 2021 Jun 30;10:97. doi: 10.1186/s13756-021-00966-z (PMC8244238; doi:10.1186/s13756-021-00966-z)
Supplement: Supplementary file 1 — Additional file 1. Questionnaire. The questionnaire represents the data collection instrument that was developed for this study, hasn’t previously been published elsewhere. [file 13756_2021_966_MOESM1_ESM.doc]

**Questionnaire of determinants of physicians’ intention to use CPGs on antimicrobials among physicians in China**

This questionnaire is being used to identify the determinants affecting physicians’ behavioral intention to use CPGs on antimicrobials. We inform you that there are no right/wrong or good/bad answers in this questionnaire; all the data we collect are confidential and for academic use only. Thank you.

**Part 1. Intention to use CPGs on antimicrobials**

Note. There are 5 numbers (1, 2, 3, 4, 5) on the right side of each item, where “1” means “Strongly disagree”, “2” means “Disagree”, “3” means “Neutral”, “4” means “Agree”, and “5” means “Strongly agree”. Please tick or circle the number that best fits your real feelings on the item.

| I am willing to use CPGs on antimicrobials. | 1 | 2 | 3 | 4 | 5 |
| --- | --- | --- | --- | --- | --- |
| I will following the CPGs on antimicrobials in the future. | 1 | 2 | 3 | 4 | 5 |
| I am willing to recommend CPGs on antimicrobials to other doctors. | 1 | 2 | 3 | 4 | 5 |

**Part 2. Potential determinants of physicians’ intentions to use CPGs on antimicrobials**

Note. There are 5 numbers (1, 2, 3, 4, 5) on the right side of each item, where “1” means “Strongly disagree”, “2” means “Disagree”, “3” means “Neutral”, “4” means “Agree”, and “5” means “Strongly agree”. Please tick or circle the number that best fits your real feelings on the item.

**Individual-level factors**

| ***Attitude*** | | | | | |
| --- | --- | --- | --- | --- | --- |
| I think it’s the right thing to follow the CPGs on antimicrobials. | 1 | 2 | 3 | 4 | 5 |
| I think it’s a wise choice to follow the CPGs on antimicrobials. | 1 | 2 | 3 | 4 | 5 |
| I think it’s good for all to follow the CPGs on antimicrobials. | 1 | 2 | 3 | 4 | 5 |

| ***Subjective norms*** | | | | | |
| --- | --- | --- | --- | --- | --- |
| People who are important to me tend to follow CPGs on antimicrobials. | 1 | 2 | 3 | 4 | 5 |
| People who are important to me have a positive evaluation of CPGs on antimicrobials. | 1 | 2 | 3 | 4 | 5 |
| People who are important to me think it’s a right thing to use CPGs on antimicrobials. | 1 | 2 | 3 | 4 | 5 |
| ***Perceived risk*** | | | | | |
| I am afraid the grasp of CPGs on antimicrobials will take extra time. | 1 | 2 | 3 | 4 | 5 |
| I am afraid prescribing via CPGs on antimicrobials will reduce revenue. | 1 | 2 | 3 | 4 | 5 |
| I am afraid prescribing via CPGs on antimicrobials will reduce efficiency. | 1 | 2 | 3 | 4 | 5 |

**Technical-level factors**

| ***Relative advantage*** | | | | | |
| --- | --- | --- | --- | --- | --- |
| Using CPGs on antimicrobials can reduce medical costs. | 1 | 2 | 3 | 4 | 5 |
| Using CPGs on antimicrobials can improve prescribing efficiency. | 1 | 2 | 3 | 4 | 5 |
| Using CPGs on antimicrobials can better clinical outcomes. | 1 | 2 | 3 | 4 | 5 |
| ***Ease of use*** | | | | | |
| Can master the knowledge of CPGs on antimicrobials in a short time. | 1 | 2 | 3 | 4 | 5 |
| Can quickly put into practice after grasping CPGs on antimicrobials. | 1 | 2 | 3 | 4 | 5 |
| It's simple and easy to use CPGs on antimicrobials in practice. | 1 | 2 | 3 | 4 | 5 |

**Organizational-level factors**

| ***Top management support*** | | | | | |
| --- | --- | --- | --- | --- | --- |
| Managers promote the wide use of CPGs on antimicrobials in various departments. | 1 | 2 | 3 | 4 | 5 |
| Managers provide supports in training, funding, etc. | 1 | 2 | 3 | 4 | 5 |
| Managers attach great importance to the promotion of CPGs on antimicrobials. | 1 | 2 | 3 | 4 | 5 |
| ***Organizational implementation*** | | | | | |
| The hospital provides information about CPGs on antimicrobials. | 1 | 2 | 3 | 4 | 5 |
| The hospital performs daily inspection, supervision and evaluation. | 1 | 2 | 3 | 4 | 5 |
| The hospital holds regular feedback on the use of CPGs on antimicrobials. | 1 | 2 | 3 | 4 | 5 |

**Part 3. Personal information card**

1. Please choose your gender.

A. Male B. Female

2. Please write down your age: ________

3. Please choose your educational Level.

A. Junior college or below B. Bachelor C. Master D. Doctor

4. Please choose your professional Title.

A. Junior B. Intermediate C. Senior

5. Please choose your working department.

A. Internal medicine B. Surgery C. Gynecology and obstetrics

D. Ophthalmology and otorhinolaryngology E. Orthopedics F. Other

6. Please choose your years in practice.

A. <5 years B. 5~10 years C. 11~15years D. 16~20 years E. >20 years
